# Frequency sensitivity in mammalian hearing from a fundamental nonlinear physics model of the inner ear

**DOI:** 10.1038/s41598-017-09854-2

**Published:** 2017-08-30

**Authors:** Karlis Kanders, Tom Lorimer, Florian Gomez, Ruedi Stoop

**Affiliations:** Institute of Neuroinformatics and Institute of Computational Science, University and ETH Zürich Irchel Campus, Winterthurerstr. 190, 8057 Zürich, Switzerland

## Abstract

A dominant view holds that the outer and middle ear are the determining factors for the frequency dependence of mammalian hearing sensitivity, but this view has been challenged. In the ensuing debate, there has been a missing element regarding in what sense and to what degree the biophysics of the inner ear might contribute to this frequency dependence. Here, we show that a simple model of the inner ear based on fundamental physical principles, reproduces, alone, the experimentally observed frequency dependence of the hearing threshold. This provides direct cochlea modeling support of the possibility that the inner ear could have a substantial role in determining the frequency dependence of mammalian hearing.

## Introduction

**Figure 1:**
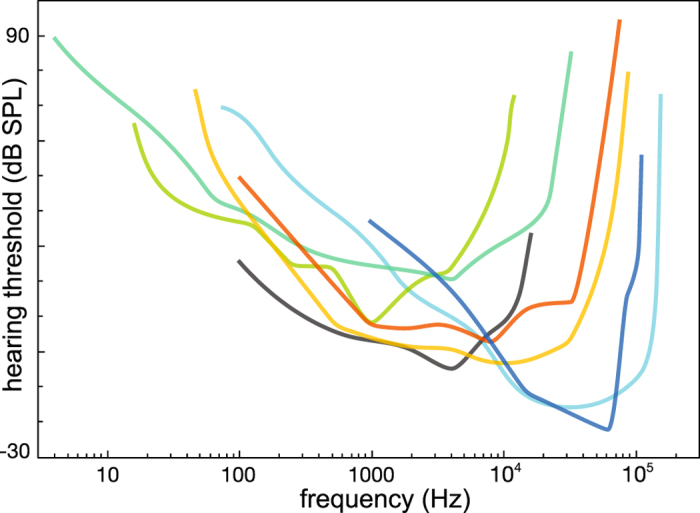
Behavioral audiograms of prairie dog^52^, elephant^53^, lemur^54^, domestic cat^55^, human psychoacoustial hearing threshold^4^, white-beaked dolphin^56^ (smoothened data), false killer whale^57^, from top to bottom, sorted by the curves’ minima. The full extension of the audiogram was not accessed in all cases. Reference sound pressure *p*_0_ = 20 *μ*Pa.

Psychoacoustic and behavioral experiments^1, 2^ exhibit a marked frequency dependence of the mammalian hearing sensitivity^3–6^ (Fig. 1). Over more than a decade, the biophysical origins of this dependence have now remained a subject of debate^3, 7–10^, despite the great development of measurement and modeling technologies. To compare directly psychoacoustic to biophysical results - which is what is generally done and what we will do below - the auditory signal would be required to remain unaltered along the auditory pathway. This is far from obvious, but recently^11^ it was shown that the auditory pathway may achieve this property by making substantial use of stochastic resonance in the auditory fibers. Here, we show that the same mesoscopic nonlinear physics model of the mammalian cochlea that has successfully reproduced other challenging hearing phenomena^12–16^ might also provide an explanation of the observed sensitivity dependence on frequency.

Let us briefly summarize the key points of controversy underlying this debate. One viewpoint emerges from following the most straightforward way of approaching this question, by conceiving the hearing sensor as composed of a resonator (outer ear), an impedance matcher (middle ear), and a Fourier analyzer of the auditory signal (inner ear), respectively (see the references given in the corresponding discussion in ref. 3). The outer ear is commonly modeled by a semi-closed cylinder^17, 18^ of about 30 mm. This leads to a resonance in the range of 3 kHz as the first, and the second one at tripled frequency; effects that are indeed observed in measured data (cf. Figs 1 and 3). The modeling of the influence of the middle ear in this process is more challenging, due to the presence of additional, complicating mechanical and spatial elements (cf. refs 19–21). The hearing pathway overall response is then obtained by performing a summation over the corresponding individual logarithmic responses. With the development of experimental techniques, this approach could be calibrated with measured animal data. In this way, qualitative aspects of the behavioral data (Fig. 1) could be reproduced for some mammals (e.g. for human hearing^10, 22^), whereas for a number of other mammals, the approach appears to have been unsuccessful so far^3^. A deviant viewpoint emerges from a more recent analysis by Ruggero *et al*.^3^, who re-evaluating middle ear transfer function data, arrived at the result that a number of animal middle ear transfer functions appear to cover a much broader interval than their actual hearing frequency interval (cf. the discussion and the corresponding Figs in ref. 3, and further refs therein). This led these authors to conclude that the inner ear could have “a crucial role in setting the frequency limits..” but that “It remains to be seen whether the finding that the bandwidth of middle-ear vibrations exceeds that of the audiogram in chinchilla, gerbil, guinea pig, horseshoe bat, pigeon, and turtle will be confirmed..”^3^. A number of subsequent biological measurements and finite element simulations seem to support the lack of frequency specificity of the outer and middle ear (e.g., refs 23, 24 and 25, respectively). For the reader’s convenience, data underlying this view are presented for the example of the Gerbil’s hearing system in our Suppl. Mat. section I. These results have, however, been challenged (cf. ref. 7 and the discussion in ref. 24); the main role in shaping hearing sensitivity seems to be commonly still attributed to the outer and middle ear.

In this dispute, regardless of which arguments will ultimately prevail, a critical analysis of the role of the active inner ear is still missing, and the analysis performed so far must thus be seen as incomplete. We contribute to the debate by investigating whether, and if so, under what conditions, an established model of the cochlea could reproduce the observed frequency dependence at all.

The biophysically detailed differential equation model of the cochlea that we use below to address this question^26^ is based on a shallow fluid wave propagating along the basilar membrane (BM), described by fluid and BM mass density, depth of the cochlear fluid canal, BM stiffness and surface tension. To these passive properties, active amplification found in the cochlea is added in the form of outer hair cell dynamics described at a mesoscopic level^26^ in terms of the Hopf small signal amplifier property^27–31^. A micro-electro-mechanical model of the embedded hair cell dynamics, that would account for the whole mammalian hearing range, is presently still missing. This, however, is not detrimental since the underlying Hopf small-signal amplifier model, which has been demonstrated to be the working principle of several animal’ hearing’ implementations (e.g., the frog’s sacculus^32–34^, or Drosophila’s antennas^35, 36^), can naturally be implemented into the biophysical framework and can directly serve for implementing the biophysically observed amplification response.

**Figure 2:**
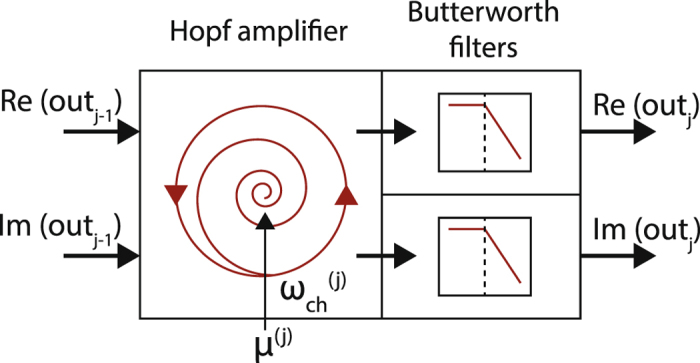
Cochlea section from a Hopf cochlea composed of several serially connected sections. A complex (real and imaginary part) signal from a precedent section (indexed by *j* − 1) enters the following section (indexed by *j*). Signal components that are close to the characteristic frequency ${\omega }_{ch}^{(j)}$ of the Hopf amplifier *j* are most strongly amplified and can be read off at this point, where the maximal amplification is determined by the Hopf parameter *μ* ^(*j*)^. Thereupon, the signal component is attenuated by a low-pass Butterworth filter, whereas sound components of lower frequencies are passed on to the next section ${\omega }_{ch}^{(j+\mathrm{1)}}$ (see text). The nonlinear nature of the amplification leads to all kinds of phenomena known from experiments on human hearing (see our Suppl. Mat. III).

Our biophysical description is of a generalist nature. It can be easily specified to implement generalist or specialist hearing properties, as well as to implement defective hearing properties (for an example, see ref. 37). Moreover, it can directly be translated into a computationally optimized form where the underlying differential equation model is discretized into a series of serially connected cochlea sections. A section, see Fig. 2, embraces the frequency-specific amplification by a patch of the basilar membrane with attached outer hair cells surrounded by the cochlear fluid. In the section, a long list of local or microscopic parameters (relevant for describing the detailed passive aspects of the cochlea or micro-electrical-mechanical of active amplification), is collapsed into two parameters per section, the Hopf bifurcation parameter *μ* (c.f. Suppl. Mat. II) and the section’s preferred frequency. In the human case (a hearing generalist, see below), the frequencies are preferably logarithmically spaced; this implementation is fully described, e.g., in the supplement of ref. 16, see also refs 12–16.

The obtained sensor has been demonstrated to reproduce all observed salient nonlinear effects of mammalian hearing, from frequency- and stimulation strength dependent amplification profiles, to the correct combination-tone generation, to the phenomena of pitch perception (pitch shift effect, loudness-dependence of pitch). Detailed comparisons of biophysical key data to the corresponding data from the Hopf cochlea, were provided in earlier publications^13, 15^, and in particular in the supplemental materials of refs 16 and 38. For convenience, a collection of salient comparisons between the biophysical data and corresponding results from our model is furnished in our Suppl. Mat. III.

Hopf amplifiers can be tuned towards higher or lower sensitivity, by adjusting their Hopf parameters closer or further away, respectively, from the Hopf bifurcation point. A species’ hearing characteristics are, at this level, characterized by a list of preferred frequencies and the sections’ “normal” sensitivities. Already in ref. 26 it was exhibited that for human-like hearing, a down-tuning of the Hopf amplifiers’ sensitivities towards more apical frequencies would be appropriate, where an analytic expression was provided. In the biological example, amplifier sensitivity can, moreover, be modulated to some extent by efferent connections. In ref. 39 (see our Suppl. Mat. II), we have shown that this property is likely a major ingredient of auditory source separation in the biological example, and how this mechanism, that turns our sensor from a “hearer” into a “listener”, can be easily implemented within the present framework. Comparative hearing research reveals for auditory specialists great frequency optimization of the BM-place-frequency map, where generalists show a much smoother variation. Both categories, however, deviate from the purely exponential dependence^40^ that would be compatible with a cochlea where all Hopf parameters have the same value (a “flat-tuned” cochlea) on a logarithmic frequency spacing. The fact that animals of variable sizes (ferret, elephant) have substantial lower hearing frequency bounds compared to humans, points to tuning as an expression of evolutionary adaptation of the different species, rather than to limitations contributed by the mechano-electrical OHC and hair bundle design. Additionally, species-specific emphasized frequency intervals may be due to neural conditioning, by which efferent connections may be designed or taught to enhance or to suppress desired and undesired, respectively, frequency ranges^39^. An observed gentle decay of the endocochlear potential along the cochlear duct is observed (which can be expected to negatively affect the efficiency of the outer hair cells (e.g., ref. 41)) and changes in the micro-mechanical properties along the cochlear duct^42^ seem, in this context, to be of secondary relevance. These mentioned observations, however, exhibit that in the context of non-local aspects of the cochlea, such as the hearing threshold’s frequency dependence across the hearing range, a tuning of the amplification along the cochlear duct must be considered. In a cochlea model based on Hopf amplifiers, ‘tuned’ amplification profiles could be obtained in two ways: by a changed density of OHC working at the same amplification strength (biologically, we observe a lowered OHC density towards the apex), or by the tuning of the sensitivity of the amplifiers. In the context of the mesoscopic approach followed in our modeling, a tuning of the sensitivity is the simpler solution. In the following, we used a tuning suggested by the estimate provided in ref. 26 that was shown to provide an accurate reproduction of Smoorenburg’s^43^ pitch shift experiments^15^. Upon such tuning, previous observations regarding local amplification, combination tone decay, phase behavior procession along the cochlea, and sound selectivity by tuning^15^, remain valid (see our Suppl. Mat. III).

To place our model into the context of psychoacoustic hearing experiments, we start from the following: if for the cochlea a Hopf parameter around $\mu \simeq -0.2$ is chosen and if about −114 dB input to the Hopf cochlea is taken to correspond to 0 dB SPL in biology^15^, the local amplification response curves of the Hopf cochlea reliably reproduce the behavior of the prominent biophysical examples obtained at high to central hearing frequencies at moderate sound pressure levels^41, 44^, given a “natural” discretization of the cochlea^16^. A 10 dB SPL input, which characterizes the hearing threshold for the most sensitive part of the human frequency range^15^, then corresponds to a −104 dB input to the Hopf cochlea, leading to a response of about −50 dB. It is therefore natural to define a node to be ‘activated’ if the amplified signal (of an arbitrary input signal), reaches at the node above a −50 dB threshold (the model’s hearing threshold). Other choices of the threshold within a ±10 dB bandwidth (including, in particular, the −53 dB value that has been used for the reproduction of the experimental data of combination-tone hearing experiments in ref. 15), did not affect any of the following results. For our experiments, we stimulated the Hopf cochlea by pure tone signals of frequency *f*, with the amplitude *A* set to a predefined level *L* in dB, i.e., *A* = 10^(*L*/20)^. Pure-tone stimulations were used as they lead to essentially one activated locus (simple non-pure stimuli lead to complex activation response patterns^16, 45^ that are more difficult to work with). From a range between −120 and −10 dB, stimulation levels were selected in steps of 1 dB, at stimulation frequencies from a (0.02,20) kHz interval, using logarithmic spacing. At each section, the response $R=20\,{\mathrm{log}}_{10}(y)$ was measured, where *y* denotes the maximal amplitude of the response obtained during the observation time. The input level for which the maximum *R*_*max*_ across all sections was closest to −50 dB, was taken as the hearing threshold input level.

**Figure 3:**
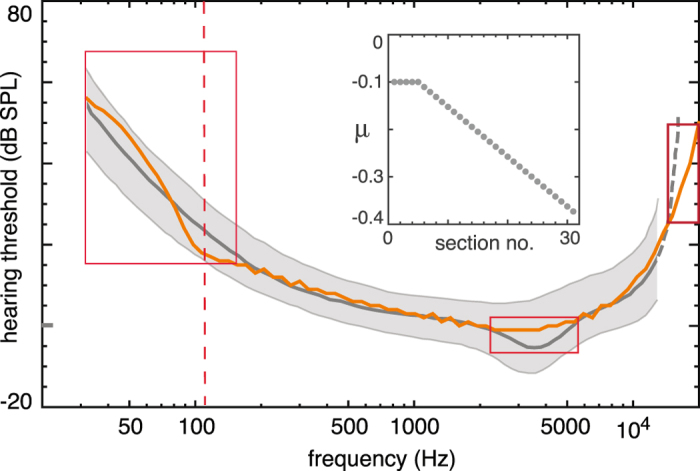
Gray curves: data from Zwicker’s publication^4, 58^, dashed part indicating extrapolations. Gray shading: observed human variability. Red curve: cochlea response with biological tuning, where the inset shows the tuning of Hopf parameters used (see text). Across sensible tuning variations, the overall behavior is remarkably stable. Three boxes indicate areas along the cochlear duct where our model deviates from the biological example (see text). Dashed vertical line: frequency location of the last section of the cochlea model.

For our main experiments, a Hopf cochlea with 31 sections, spanning 110–19912 Hz, tuned at Hopf parameters *μ* at −0.1 for the first few sections (coding for the highest frequencies), followed by a steady decrease of −0.0105 per section, was used (the latter value depends on the particular discretization). Also neighboring configurations were tested (e.g. where an immediate decay was implemented); all of them reproduced the experimental audiogram from ref. 4 remarkably well (c.f. Fig. 3).

**Figure 4:**
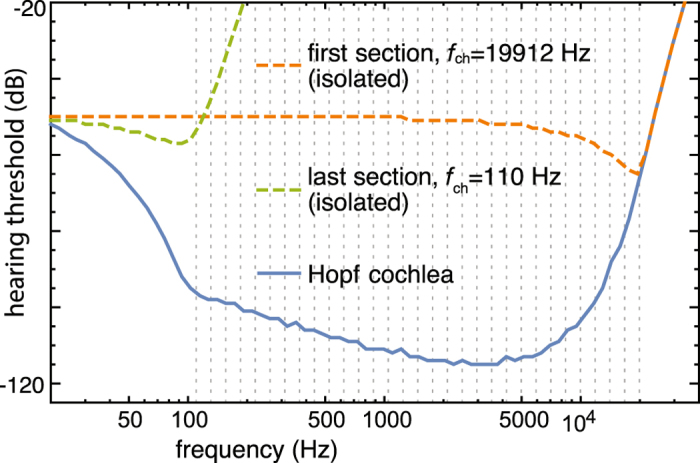
Sensitivity at the borders of the frequency hearing interval exhibiting that the first section determines the high-frequency response. The response at the low-frequency border, however, emerges as a cumulative, collective result that will also be influenced by boundary and discretization effects. Dashed vertical lines indicate the locations of the Hopf amplifiers’ characteristic frequencies.

**Figure 5:**
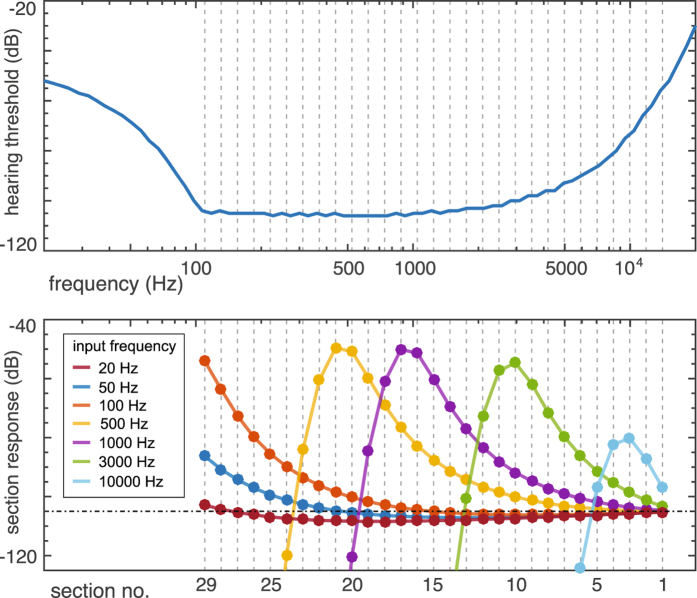
Mechanism underlying frequency dependence of the hearing threshold. Lower panel: evolution of the amplification profile along the cochlear duct, for seven input frequencies. Upper panel: obtained hearing threshold. For clarity of the effect, a flat-tuned cochlea of 29 sections was used. Horizontal dashed-dotted line: input amplitude.

The deviations between psychoacoustic measurements and estimates and our simulation results have the following natural explanations (Fig. 3, boxes from right to left). At the highest frequencies, a precipitous rise of the hearing threshold curve at the base has been estimated by ref. 4 (first box, dashed line). Our analytic model of the passive model of the basilar membrane^26^ corroborates that salient effects of the BM attachment are basal to the characteristic locus of our first amplifier (if reliable biophysical data were available, our computational approach could be adapted to take such data into account as well (cf. first box and Fig. 4)). After exploiting the full interaction range - at the given discretization and sensitivity of our Hopf cochlea, this influence extends over a range of roughly 6–8 sections (cf. Fig. 5, lower panel) - a region of optimal sensitivity is reached where the behavior predicted by our model deviates from the biological example (second box). This deviation is due to the well-known resonance effect by the outer and the middle ear, known to contribute a stimulation increase effect up to 10 dB, confined to the area of interest around 4000 Hz^18^. Taking this into account yields a very good agreement with the biological example. Further towards the apex the sensitivity slowly decreases, due to the decrease in Hopf sensitivity. Beyond the characteristic frequency of the last amplifier section, our model deviates from the example as the discretization of our model fails to account for the more gradual amplification decrease of the biological example (indicated by a reduced number of OHC-rows), third box. Beyond the characteristic frequency of the last amplifier, the amplification vanishes therefore more abruptly than in the biological example, and it is also known that towards the apex, the membrane vibrations are less sharply tuned^46^. Toward the physical end of the basilar membrane, also other salient effects are neglected, in particular the vanishing pressure difference between the scala media and the scala tympani that must be expected to have a strong effect on the biological response. We suspect that if that frequency region could be accessed experimentally (a quite difficult endeavor^47^), a precipitous rise of the curve would be observed. Reliable data, however, are sparse also in this case.

The above interpretation can be corroborated by analytical considerations that, moreover, nicely exhibit the fundamental mechanism by which the frequency dependence of the hearing threshold is obtained in our model. The complex-number normal form Hopf equation, rescaled by a characteristic angular frequency *ω*_*ch*_ and subjected to a sinusoidal forcing signal, $\dot{z}={\omega }_{ch}((\mu +i)z-{|z|}^{2}z+F{e}^{i\omega t})$, $z\in {\mathbb{C}}$, can be rewritten in a coordinate frame that rotates with the forcing: $\dot{z}={\omega }_{ch}(\mu z-{|z|}^{2}z+F)+i({\omega }_{ch}-\omega )z\,$. Assuming after some transient period a 1:1 locked solution (existence verified in the numerical experiments), *z*(*t*) = *ae* ^*iϕ*^ is constant in this coordinate frame, and one can obtain a cubic polynomial in *a* ^2^ that can be solved for the real amplitude *a* ^12, 30, 45^. Alternatively, one can separate the locking criterion into two conditions $\dot{a}=\mu a-{a}^{3}+F\,\cos \,\varphi =0$, and $\dot{\varphi }=({\omega }_{ch}-\omega )a-{\omega }_{ch}F\,\sin \,\varphi =0$. The first condition has no direct dependence on *ω*, but from the second, assuming $|\varphi |\to \tfrac{\pi }{2}$ for large |*ω*_*ch*_ − *ω*|, we can approximate

1 $$a\approx \frac{{\omega }_{ch}F}{|{\omega }_{ch}-\omega |},\,{\rm{for}}\,|{\omega }_{ch}-\omega |\gg 0.$$

This approximation, which shows good agreement with the full analytic solution for small negative *μ* and small forcing *F* (cf. Suppl. Mat. II), provides a clear picture of the collective amplification that is responsible for the pure-tone hearing threshold curve. To focus and separate influences, we illustrate our arguments using a flat-tuned Hopf cochlea (i.e., a cochlea for which all Hopf parameters are all at the same value, *μ*_*i*_ = const., *i* = 1 … 29). For input frequency $\omega \ll {\omega }_{ch}$, we have a Hopf response amplitude *a* ≈ *F*. This defines a natural limit for the number of higher frequency Hopf cochlea sections that most significantly influence the amplification cascade of an input signal (in our setting, roughly the mentioned 6–8 sections, cf. Fig. 5). Over the frequency range covered by those high-frequency sections, the hearing threshold decreases, due to the onset of the amplification cascade. After the interaction range is exploited, the maximum hearing sensitivity is reached, and a flat hearing threshold curve is maintained until the characteristic frequency of the last section is reached. After the last cochlea section, as the input frequency moves beyond the amplification envelope of the last cochlea sections, the hearing threshold increases again. This also implies that for very low frequency, the input threshold of the Hopf cochlea approaches, in the model, the pre-specified output threshold of −50 dB (i.e., *a* ≈ *F*).

In this way, essential frequency dependence of the hearing threshold emerges from a neighborhood preceding and including the signal frequency’s best matching amplifier. This neighborhood delivers the principal part of the amplification, where the asymmetry in frequency (cf. Fig. 3, Suppl. Mat. III) is the consequence of the Butterworth filter. The obtained amplification can be modified by a tuning of the Hopf amplifiers’ sensitivity along the cochlear duct, such as is the case in human hearing, where we observe a simple continued down-regulation of the sensitivity. On the high frequency branch, amplification accelerates by recruiting ever more amplifiers of this neighborhood, until the maximal neighborhood range is reached. After this point, the slow detuning of the amplifiers’ efficiency becomes effective, until the signal frequency is below the characteristic frequency of the last amplifier. Here, a de-recruitment process sets in, where only the remaining amplifiers of the neighborhood - at non-optimal characteristic frequencies - still contribute to the amplification. As our system is based on a detailed model of the inner ears’ physical properties, we expect this explanation to hold for the biological example as well.

While the questions of how the microscopic mechano-electromotile systems co-operate to arrive at the Hopf property^47–49^ and how this translates to the mesoscopic approach^50^ are of great interest themselves, our mesoscopic cochlea model embraces both effects within its Hopf section. The model reproduces to great accuracy not only the naturally measured local nonlinearities^13, 38^ and ensuing salient nonlinear phenomena of hearing (such as the perception of pitch), but also the measured psychoacoustic hearing threshold’s dependence on frequency. On this mesoscopic level, we see strong evidence for a potential essential role of the inner ear in determining the sensitivity dependence of mammalian hearing. While our model is composed of identical amplifiers (modulo their characteristic frequencies and *μ*-values), it is their nonlinear interaction that binds the cochlea towards having its specific, biophysically and psycho-acoustically observed frequency-specific sensitivity. This uncovered feature is, on a more global scale of the cochlea, an even more fundamental manifestation of nonlinearity in hearing, compared to the fact that for signals composed of different frequencies, combination tone avalanches emerge (cf. ref. 16). Our results also highlight a strong role of nonlinear dynamics in explaining and reproducing biological nature: in our model, the same nonlinear principle, expressed in its most simple form, reproduces without requiring any additional efforts a whole spectrum of previously unexplained salient hearing phenomena (see our Suppl. Mat. III). This has a number of advantages, in abstract modeling and also when it comes to engineering implementations. To arrive at an amplification of incoming sound at any desired level, only a change of the Hopf parameter is required, with a much reduced latency (compared to the activation of a cascade of linear amplifiers, where the amplification strength would be naturally bounded by the number of elements of the cascade). Such tuning is, in particular, fruitful for the implementation of the role of the biological efferent connections to the cochlea. These connections are likely involved in the enhancement of desired auditory objects from an auditory landscape, by the enhancement of the corresponding frequency channels^39^ (although we deal with a nonlinear system). On a more general level, the approach taken here may open up new perspectives for research and engineering in the field of sensors. As one example, our analytical approximation to the full nonlinear-physics based model of the cochlea, reveals “filters” of a very particular form and enormous spatially extended overlaps. A classical engineer would hardly come up with such a design that poses a significant paradigm challenge to the conventional state of art. In the field of hearing, our modeling approach might provide insight into questions that are inaccessible to current experimental techniques, by guiding towards the formulation of closely related problem statements that can be experimentally accessed.

The question whether the outer-middle or the inner ear provides the limiting factor of the hearing, might, in our view, be wrongly posed after all. If we admitted that the inner ear is sufficient for explaining frequency sensitivity, could we conclude that the outer and middle ear have (apart from the already mentioned resonances) a minor role in shaping the hearing threshold? Obviously, evolution may occasionally have shaped ear canal and outer ear resulting in a constraint of hearing sensitivity. However, even the evolutionary adaption from today’s land-based towards water-based mammals did not substantially affect the cochlear construction principle^36, 51^, while it clearly affected the outer ear. In this light, while the inner ear should not be seen as the only relevant part, it seems that, as the evolutionarily less variable construction, it has the dominant role for shaping the hearing threshold. In either case, our results strongly suggest that the various statements in textbooks exhibiting that hearing sensitivity is generally determined by the outer and middle ear (but not by the inner ear), may be historically biased and not be fully justified.

## Electronic supplementary material

Supplement
