# Supplementary material for: Frequency sensitivity in mammalian hearing from a fundamental nonlinear physics model of the inner ear
Source: Sci Rep. 2017 Aug 30;7:9931. doi: 10.1038/s41598-017-09854-2 (PMC5577103; doi:10.1038/s41598-017-09854-2)
Supplement: Supplementary file 1 — Supplement [file 41598_2017_9854_MOESM1_ESM.pdf]

Supplemental materials to "Frequency sensitivity in mammalian hearing from a fundamental nonlinear physics model of the inner ear", by Kanders, K., Lorimer, T., Gomez, F. & Stoop, R.

I: MONGOLIAN GERBIL FREQUENCY HEARING CHARACTERISTICS

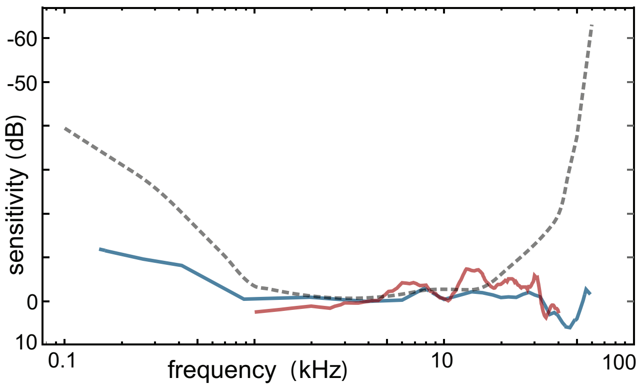

FIG. 1: Outer-middle ear transfer functions of the Mongolian gerbil that serves in a wide range as a model of mammalian hearing. Dashed: Behavioral hearing threshold [1]. Curves normalized to the corresponding measurement values at 4 kHz. Blue: Pressure in scala vestibuli near stapes footplate P_{SV} [2]. Red: Stapes velocity V [3]. Original data read off from the original publications and put together by the present authors.

Fig. 1 collects some of the data (for the example of the Mongolian Gerbil) [4], that let Ruggero et al. and colleagues conclude that the inner ear could have a more substantial role in shaping the frequency sensitivity of the mammalian hearing system. A number of newer biological measurements and finite element simulations seem to support the lack of frequency specificity of the outer and middle ear (e.g., [5, 6], and [7], respectively). For the reader's convenience, data underlying this view are presented for the example of the Gerbil's hearing system.

II: METHODS

Over varying species-specific frequency intervals, mammalian hearing is able to access a huge dynamic range of sound (between 120-130 dB). This is due to the ability of the cochlea's outer hair cells to generate nonlinear amplification of the signal, leading to strong amplification of weaker sounds and weaker amplification of stronger sounds [8]. Outer hair cells follow in physical space and in frequency space (connected by the tonotopic map) a largely scaled building plan [9]. The mammalian hearing sensor, the cochlea, can therefore be described at several levels. The finest one is the level of the outer hair cells, focusing on explaining the intriguing interaction between hair bundle mechanics and electromotility of the hair cell bodies. On a more mesoscopic level, the cochlea's building plan can be captured in terms of so-called Hopf amplifier systems (e.g. [9]) that are composed as a sequence of mesoscopic sections representing discretization parts of the cochlea towards the Hopf cochlea (e.g. [10]). The device reflects the biophysics, including hair cell, basilar membrane, and fluid properties of the real cochlea in one model, to such an extent that all salient measured properties in biology could be verified in corresponding simulations. The composition of such sections into a macroscopic model of the sensor [11–13] is based on the detailed biophysics and nonlinear dynamics at work in the cochlea [14, 15]. Fundamental for this model is that the sections share the dynamical properties of the microscopic amplification-providing outer hair cells [15, 16], which are well-modeled by a stimulated *Hopf* process

$$\dot{z} = \omega_{ch}((\mu + i)z - |z|^2 z - F(t));\ z,\ F(t) \in \mathbb{C},$$

where $z(t)$ denotes the response amplitude, $F(t)$ a stimulation signal, ω_{ch} is the characteristic frequency of the Hopf system, and μ is the *Hopf parameter* [14, 15, 17–19]. At values $\mu < 0$, the system is below bifurcation to self-oscillation, but responds towards stimulation signals $F(t)$ as a small-signal amplifier [20–22]. Dissipation by fluidal viscous losses can be described by tailored 6th-order Butterworth low-pass filters [10, 12]. The main characteristics of the isolated node dynamics are collected in Ref. [19]. When embedded into a compound cochlea, the response profiles broaden due to the sections' interaction with neighboring ones, reproducing the biological data [23] extremely well [10]. The distance of μ from bifurcation at $\mu = 0$ defines how strongly a node amplifies an incoming signal; we choose this parameter to match the human hearing sensor. The biophysical properties of the cochlea suggest selecting the characteristic frequencies of the nodes according to a geometric sequence. We use a software implementation of an earlier hardware realization of 29 sections or nodes, taking care of 7 octaves (14.08 − 0.11 kHz), or a 31-section model covering an interval of (19.912 − 0.11) kHz. Our partition is optimal in the sense that finer partitions yield for the human amplification range, identical results, but coarser partitions lead to distortions in the frequency dependence, if sufficiently strong amplification is required. For *'flat tuning'*, $\mu \equiv$ const, all nodes have identical Hopf parameters (conventionally $\mu \equiv -0.25$) [19], unless we provide them with a soft continued gradual amplification decay towards lower frequencies, to optimally match the human hearing system, or if we tune them actively (mimicking the effect of efferent cochlear connections) in the context of *learning* [13]. In the latter case, we condition the network towards chosen sounds by tuning unsuited nodes towards weaker amplification. A detailed account on the design of the used cochlea is provided in the supplements of Ref. [19].

Analytical approximation of the Hopf amplification

In Fig. 2, we demonstrate the essentials of the approximation by Eq.(1) in the main text.

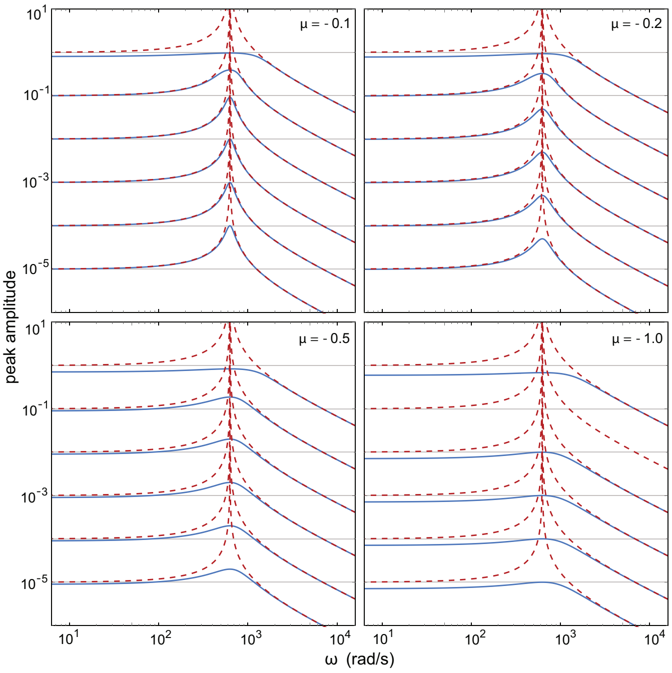

FIG. 2: Response of a single ω-rescaled Hopf system (solid blue) and the approximation given in Eq. (1) of the main manuscript (dashed red), to pure tone input of peak amplitude 10^0, 10^{-1}, 10^{-2}, 10^{-3}, 10^{-4}, 10^{-5} (top to bottom), for values of μ as indicated. For low amplitude input, and μ close to zero, the approximation shows good agreement away from the characteristic frequency of the Hopf system.

III: COMPARISON BIOLOGY-MODEL

Introduction

We exhibit here the comparison between our developed cochlea model and the corresponding biological measurements. The hearing threshold involves the whole extension of the cochlea. Across this distance, a detuning becomes essential. Almost all comparisons made below refer, however, to local properties (involving neighboring sections only). Across this distance, the detuning is without a noticeable effect. Exceptions to this statement are Figs. 8, 10 and 13 of this section, where the measurements are from an appropriately adapted cochlea.

Left hand side panels are normally the biophysical measurements, right hand side panels the corresponding modeling data. Modeling data are either from a hardware implementation of the model (Figs. 3b) - 7b), 9b)), or real-time computational (Figs. 8b), 10a),b), 11, 12, 13a),b)). These results indicate the level of reliability of the model in describing the biophysical processes.

Note that our cochlea model focuses on the active amplification by the inner ear. In addition to active amplification, other processes influence hearing, in particular at high SPL (e.g., bone conductance) or at low frequencies (vibrotactile excitation). These effects are not the subject of our model.

Local amplification

Originally, our model of the cochlea was tuned to match the measured amplification profile of the chinchilla cochlea, one of the best-studied biological cochleas.

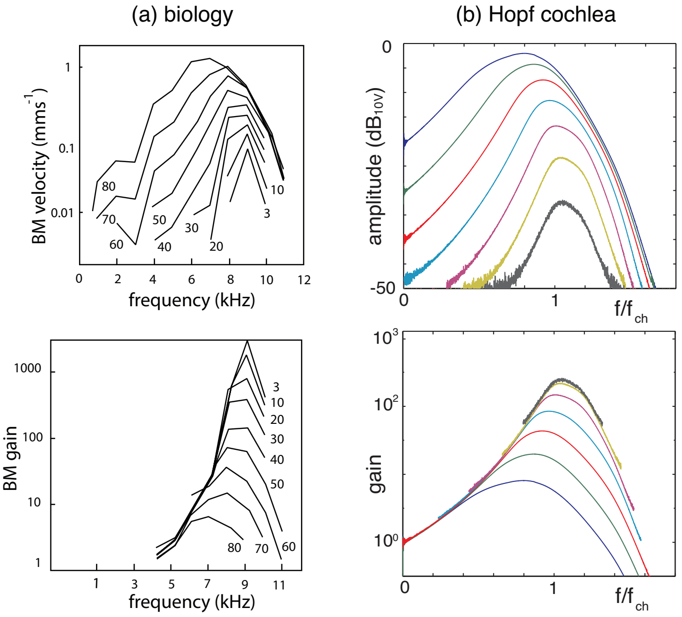

FIG. 3: Steady state response (top) and gain (bottom) for the biological example (chinchilla, f_{ch} = 9 kHz; adapted from Ref. [23]) and Hopf cochlea (f_{ch} = 5.92 kHz, μ=-0.1) [11].

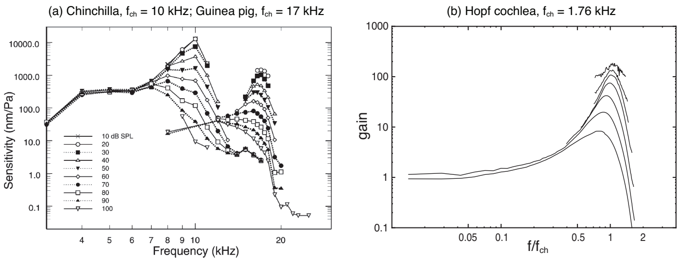

FIG. 4: Single tone gains. Gain lines representing iso-intensity stimulation curves, measured at a place along the cochlea of preferred stimulation frequency f_{ch} for (a) two mammalian species [24] (see also see also [8, 25]), where the basilar membrane displacement relative to the sound pressure level at the eardrum was measured; (b) Hopf cochlea [12].

Compression

Compression of strong inputs is one characteristic nonlinear feature of the mammalian cochlea. Particularities of the compression also justify the usage of the Hopf small signal amplifier as the underlying amplification concept.

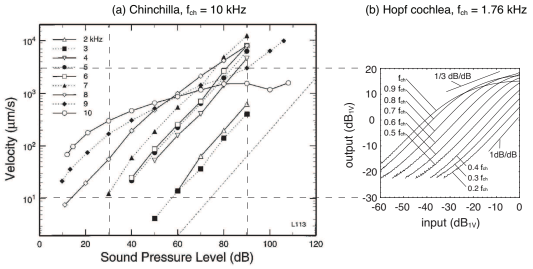

FIG. 5: Iso-frequency input-output characteristics for (a) chinchilla [8], (b) Hopf cochlea [12].

Two-tone suppression

Mutual compression of neighboring tones is another nonlinear feature of human hearing. The effect can be considered as a prototype of computation done by the mammalian hearing sensor [26].

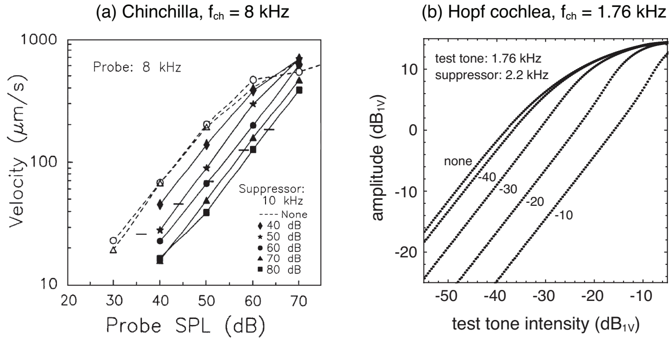

FIG. 6: Mutual suppression by two neighboring tones (‘test tone’ and ‘suppressor tone’), as a function of their intensity: (a) chinchilla [27], (b) Hopf cochlea [12].

Combination tones

The nonlinearities in the amplification process also introduce, by means of amplifier interaction, additional tones called combination tones. Such tones, and in particular their decay laws, are of great importance for the human perception of pitch.

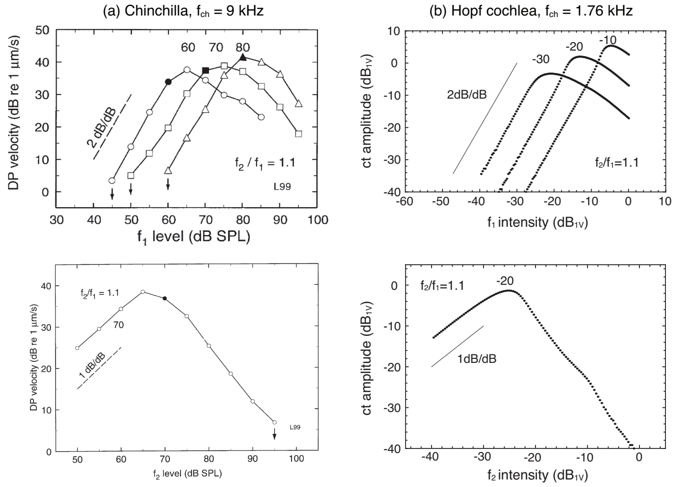

FIG. 7: Amplitude of combination tones (‘ct’) generated by simultaneous stimulation with two pure tones for (a) chinchilla [28], (b) Hopf cochlea [12]. Top panels: dependence on f_1; bottom panels: dependence on f_2.

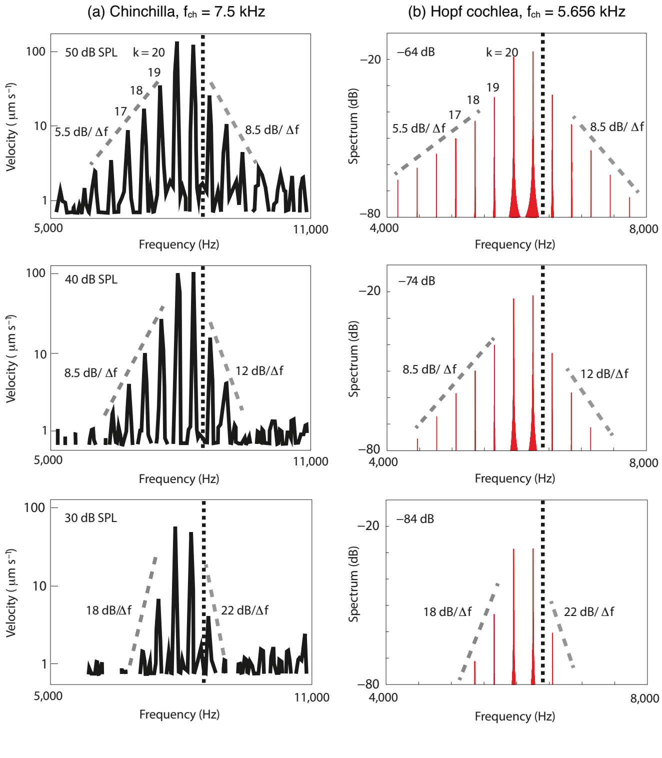

FIG. 8: Basilar membrane response spectrograms for two-tone stimulation of amplitudes 30, 40, 50 dB sound pressure level (SPL) (frequencies $f_2/f_1 = 1.05$ and $2f_2 - f_1 = f_{ch}$) for (a) chinchilla (f_{ch} = 7.5 kHz) [28], (b) Hopf cochlea, 6th section (f_{ch} = 5.656 kHz) [29]. Grey dashed lines: exponential amplitude scaling ($\Delta f = f_2 - f_1$).

Phase characteristics

The phase behavior along the cochlea also follows that in the biological example.

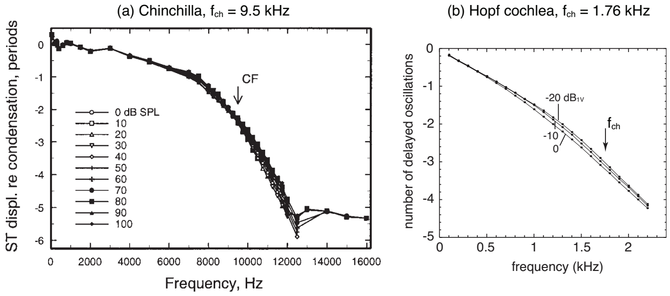

FIG. 9: Phase propagation along the cochlea: (a) chinchilla [30], (b) Hopf cochlea [12].

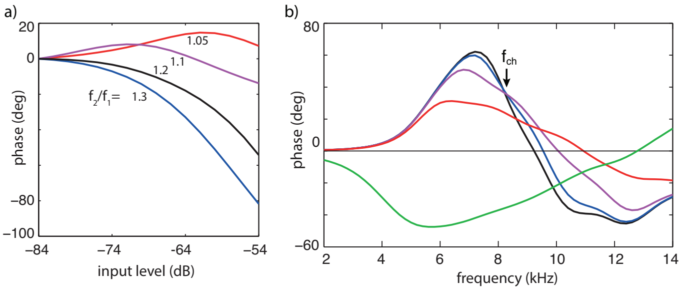

FIG. 10: Phase characteristics of the Hopf cochlea [29]: (a) Phase of the $2f_1 - f_2$ combination tone as a function of input sound level (normalized to phase at -84 dB) for four different f_2/f_1-ratios with $2f_1 - f_2 = f_{ch}$ (5th section, f_{ch} = 6.79 kHz); (b) Phase for pure-tone stimulation of various frequencies and input sound levels -74, -64, -54, -44, -24 dB (black, blue, magenta red, green) at the 4th cochlea section (f_{ch} = 8.15 kHz), normalized to the phase at -34 dB input level.

Medial efferent inhibition

The effect of a tuning of the cochlea by efferent medial olivocochlear stimulation has also been compared.

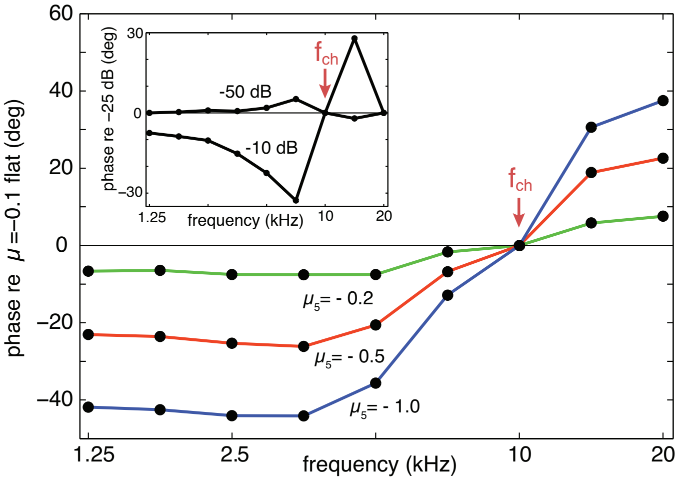

FIG. 11: Effect on phase of medial olivocochlear efferent stimulation, modelled as detuning of μ [13]. Phase shift at 5th section (f_{ch} = 10.42 kHz), μ_5 is tuned away from flat tuning $\mu = -0.1$ (18 sections, range 20 to 1.25 kHz, stimulation at -25 dB). Phase delays result for frequencies below f_{ch}, phase leads above f_{ch}. Inset: Phase level dependence relative to -25 dB (single oscillator, f_{ch} = 10 kHz, $\mu = -0.1$). Decreased low-frequency input levels lead to small phase leads; increased input levels lead to phase lags.

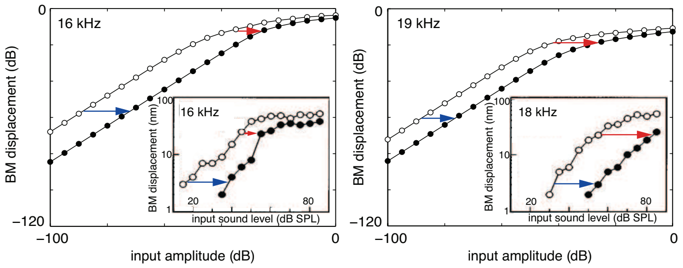

FIG. 12: Basilar membrane shifts (arrows) at 2nd section, f_{ch} = 16.99 kHz (18 sections, 20 − 1.25 kHz), when stimulated by a 16 and 19 kHz (left and right) pure tone. Open circles: Flat tuning ($\mu = -0.05$). Filled circles: medial olivocochlear efferent stimulation; μ is shifted to −0.5. Insets: Corresponding animal data [31].

Pitch shift

Another characteristic feature of the biological cochlea is the so-called pitch shift effect. The effect describes the human perception of pitch when stimulated by two tones, one of which is shifted in frequency with respect to the other's frequency.

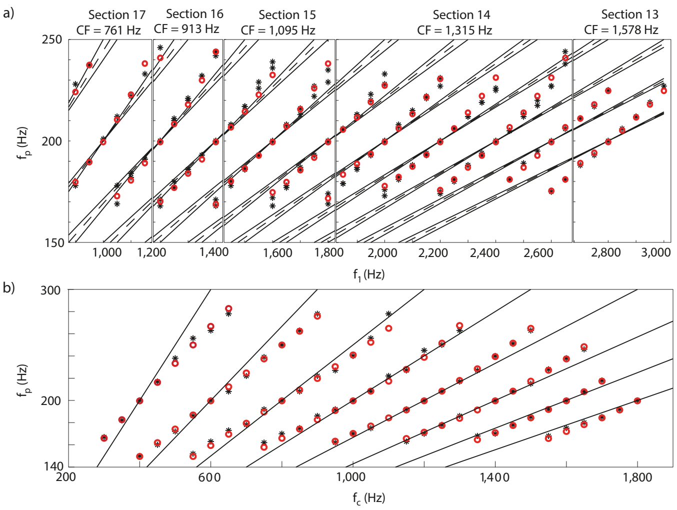

FIG. 13: Pitch-shift experiment [29]. (a) Two-frequency stimulation $f_2 = f_1 + 200$ Hz. Black stars: psychoacoustic data [32] (partial sound levels 40 dB sound pressure level, two subjects). Red circles: Hopf cochlea (sections as indicated, tones at -74 dB each). Black lines: false predictions by de Boer's formula [33] for $k' = k$, $k' = k + 1/2$ (dashed) and $k' = k + 1$, respectively. (b) Response of a cell of the cat ventral nucleus [34] ('On-L-cell', $f_{ch} = 1.1$ kHz) to a three-frequency stimulation ($(f_c - f_{mod})$, f_c , $(f_c + f_{mod})$; $f_{mod} = 200$ Hz) at 50 dB SPL. Black stars: inverse of the most frequent interspike intervals. Red circles: pitch from 15th cochlea section ($f_{ch} = 1.095$ kHz, -64 dB).

[1] Ryan, A. Hearing sensitivity of the mongolian gerbil, Meriones unguiculatis. *J. Acoust. Soc. Am.* **59**, 1222-1226 (1976).

[2] Olson, E.S. Intracochlear pressure measurements related to cochlear tuning. *J. Acoust. Soc. Am.* **110**, 349-367 (2001).

[3] Overstreet, E.H. III & Ruggero, M.A. Development of wide-band middle ear transmission in the Mongolian gerbil. *J. Acoust. Soc. Am.* **111**, 261-270 (2002).

[4] Ruggero, M.A. & Temchin, A.N. The roles of the external, middle, and inner ears in determining the bandwidth of hearing. *Proc. Natl. Acad. Sci. U.S.A.* **99**, 13206-13210 (2002).

[5] Zosuls, A., Mountain, D.C. & Ketten, D.R. How is sound conducted to the cochlea in toothed whales? *AIP Conf. Proc.* **1703**, 060006 (2015).

[6] Robles, L., Temchin, A.N., Fan, Y. & Ruggero, M.A. Stapes vibration in the chinchilla middle ear: Relation to behavioral and auditory-nerve thresholds. *J. Assoc. Res. Otolaryngol.* **16**, 447-457 (2015).

[7] Yao, W., Ma, J. & Huang, X. Numerical simulation of the human ear and the dynamic analysis of the middle ear sound transmission. *J. Instrum.* **8**, C06009 (2013).

[8] Ruggero, M.A., Rich, N.C., Recio, A., Narayan, S.S. & Robles, L. Basilar membrane responses to tones at the base of the chinchilla cochlea. *J. Acoust. Soc. Am.* **101**, 2151-2163 (1997).

[9] Lorimer, T., Gomez, F. & Stoop, R. Mammalian cochlea as a physics guided evolution-optimized hearing sensor. *Sci. Rep.* **5**, 12492 (2015).

[10] Martignoli, S., van der Vyver, J.-J., Kern, A, Uwate, Y. & Stoop, R. Analog electronic cochlea with mammalian hearing characteristics. *Appl. Phys. Lett.* **91**, 064108 (2007).

[11] Stoop, R., Jasa, T., Uwate, Y. & Martignoli, S. From hearing to listening: Design and properties of an actively tunable electronic hearing sensor. *Sensors* **7**, 3287-3298.

[12] Martignoli, S. & Stoop, R. Local cochlear correlations of perceived pitch. *Phys. Rev. Lett.* **105**, 048101 (2010).

[13] Gomez, F., Saase, V., Buchheim, N. & Stoop, R. How the ear tunes in to sounds: A physics approach. *Phys. Rev. Applied* **1**, 014003 (2014).

[14] Kern, A. & Stoop, R. Essential role of couplings between hearing nonlinearities. *Phys. Rev. Lett.* **91**, 128101 (2003).

[15] Kern, A. *A nonlinear biomorphic Hopf-amplifier model of the cochlea*. PhD thesis, ETH Zurich (2003).

[16] Gomez, F., Lorimer, T. & Stoop, R. Signal-coupled subthreshold Hopf-type systems show a sharpened collective response. *Phys. Rev. Lett.* **116**, 108101 (2016).

[17] Eguiluz, V.M., Ospeck, M., Choe, Y., Hudspeth, A.J. & Magnasco, M.O. Essential nonlinearities in hearing. *Phys. Rev. Lett.* **84**, 5232 (2000).

[18] Camalet, S., Duke, T., Jülicher, F. & Prost, J. Auditory sensitivity provided by self-tuned critical oscillations of hair cells. *Proc. Natl. Acad. Sci. U.S.A.* **97**, 3183-3188 (2000).

[19] Stoop, R. & Gomez, F. Auditory power-law activation avalanches exhibit a fundamental computational ground state. *Phys. Rev. Lett.* **117**, 038102 (2016).

[20] Wiesenfeld, K. & McNamara, B. Period-doubling systems as small-signal amplifiers. *Phys. Rev. Lett.* **55**, 13 (1985).

[21] Derighetti, B., Ravani, M., Stoop, R., Meier, P.F., Brun, E. & Badii, R. Period-doubling lasers as small-signal detectors. *Phys. Rev. Lett.* **55**, 1746 (1985).

[22] Wiesenfeld, K. & McNamara, B. Small-signal amplification in bifurcating dynamical systems. *Phys. Rev. A* **33**, 629 (1986).

[23] Ruggero, M.A. Responses to sound of the basilar membrane of the mammalian cochlea. *Curr. Opin. Neurobiol.* **2**, 449-456 (1992).

[24] Robles, L. & Ruggero, M.A. Mechanics of the mammalian cochlea. *Physiol. Rev.* **81**, 1305-1352 (2001).

[25] Cooper, N.P. & Rhode, W.S. Mechanical responses to two-tone distortion products in the apical and basal turns of the mammalian cochlea. *J. Neurophysiol.* **78**, 261-270, (1997).

[26] Stoop, R. & Kern, A. Two-tone suppression and combination tone generation as computations performed by the Hopf cochlea. *Phys. Rev. Lett.* **93**, 268103 (2004).

[27] Ruggero, M.A., Robles, L. & Rich, N.C. Two-tone suppression in the basilar membrane of the cochlea: Mechanical basis of auditory-nerve rate suppression. *J. Neurophysiol.* **68**, 1087-1099 (1992).

[28] Robles, L., Ruggero, M.A. & and Rich, N.C. Two-tone distortion on the basilar membrane of the chinchilla cochlea. *J. Neurophysiol.* **77**, 2385-2399 (1997).

[29] Gomez, F. & and Stoop, R. Mammalian pitch sensation shaped by the cochlear fluid. *Nat. Phys.* **10**, 530-536 (2014).

[30] Ruggero, M.A., Narayan, S.S., Temchin, A.N. & Recio, A. Mechanical bases of frequency tuning and neural excitation at the base of the cochlea: Comparison of basilar-membrane vibrations and auditory nerve-fiber responses in chinchilla. *Proc.*

Natl. Acad. Sci. U.S.A. **97**, 11744-11750 (2000).

[31] Russell, I.J. & and Murugasu, E. Medial efferent inhibition suppresses basilar membrane responses to near characteristic frequency tones of moderate to high intensities. *J. Acoust. Soc. Am.* **102**, 1734-1738 (1997).

[32] Smoorenburg, G.F. Pitch perception of two-frequency stimuli. *J. Acoust. Soc. Am.* **48**, 924-942 (1970).

[33] de Boer, E. On the 'residue' and auditory pitch perception in *Auditory System Vol. 3 (Handbook of Sensory Physiology)* (ed. Keidel, W.D. & Neff, W.D.), pp. 479-583, (Springer, Berlin, 1976).

[34] Rhode, W.S. Interspike intervals as a correlate of periodicity pitch in cat cochlear nucleus. *J. Acoust. Soc. Am.* **97**, 2414-2429 (1995).
